# Clinical and radiologic criteria to predict endoscopic third ventriculostomy success in non-communicating pediatric hydrocephalus

**DOI:** 10.1007/s00381-024-06704-1

**Published:** 2024-12-16

**Authors:** Davit Tatoshvili, Andreas Schaumann, Anna Tietze, Valentina Pennacchietti, Gesa Cohrs, Matthias Schulz, Ulrich-W. Thomale

**Affiliations:** 1 Pediatric Neurosurgery, Charité - Universitätsmedizin Berlin, Campus Virchow Klinikum, Augustenburger Platz 1, 13353 Berlin, Germany; 2 Charité - Universitätsmedizin Berlin, Institute of Neuroradiology, Augustenburger Platz 1, 13353 Berlin, Germany

**Keywords:** Neuroendoscopy, Third ventriculocisternostomy, ETV, Non-communicating hydrocephalus, Shunt independence

## Abstract

**Objective:**

Endoscopic third ventriculocisternostomy (ETV) became the relevant treatment option for non-communicating pediatric hydrocephalus. ETV success was predicted in relation to age, diagnosis, and previous shunt implantation. Radiological factors are usually taken for indication decision-making. The aim of this study is to investigate radiological signs of non-communicating hydrocephalus for ETV success in a single-center retrospective analysis.

**Patients and methods:**

ETV interventions were collected from a 10-year period (2010–2019) from our institution. Clinical patient characteristics such as prematurity, age, diagnosis, and previous shunt treatment and follow-up in terms of possible shunt implantation or revision surgeries were investigated. Radiological data was retrieved from the in-house PACS system to analyze preoperative signs for non­communicating hydrocephalus such as ventricular size, pressure gradients at the third ventricle, and any signs of obstruction from internal towards external cerebral spinal fluid communication. Fisher’s test was used to demonstrate the significance of each individual predictor. A multivariable model was built using the backward elimination method with multiple logistic regression.

**Results:**

From 136 ETV interventions, 95 met the inclusion criteria (age < 18 years; > 6-month follow-up; MR image data availability, treatment goal for shunt independence). In chi-square statistical evaluation of single parameters age > 6 months (OR 32.5; 95% CI 4.8–364), ventricular width (FOHR < 0.56; OR 6.1; 95% CI 2.2–16.3) and non-post-hemorrhagic hydrocephalus as underlying diagnosis (OR 13.1; 95% CI 1.9–163) showed significant increased odds ratio for shunt independence during follow-up. Logistic regression analysis for multiple parameters showed age > 6 months (OR 29.3; 95% CI 4.1–606) together with outward bulged lamina terminalis (OR 4.6; 95% CI 1.2–19.6), smaller FOHR (continuous parameter; OR 2.83 × 10^−5^; 95% CI 4.7 × 10^−9^–0.045), and non-4th-ventricular-outlet obstruction (4thVOO; OR 0.31; 95% CI 0.09–1.02) as significant factors for ETV success.

**Conclusion:**

ETV has become a relevant treatment for non-communicating hydrocephalus, with typical MR image characteristics. Analyzing radiological markers as predictors for success smaller ventricular width and outward displaced lamina terminalis was relevant in combination with age > 6 months. Since the analysis is based on single-center experience, a larger cohort of patients with a multi-center approach should further investigate the combined clinical and radiological criteria.

**Supplementary Information:**

The online version contains supplementary material available at 10.1007/s00381-024-06704-1.

## Introduction

Hydrocephalus, a neurological condition characterized by abnormal cerebrospinal fluid (CSF) dynamics, poses significant health challenges worldwide. This condition can lead to increased intracranial pressure and brain damage, leading to possible impairment in neurocognitive and motor development, seizures, spasticity, and mortality. It is estimated that there are nearly 400,000 new cases of pediatric hydrocephalus each year worldwide [1]. Among other procedures, endoscopic third ventriculostomy (ETV) has emerged as a valuable neurosurgical procedure for the treatment of non-communicating hydrocephalus, providing a less invasive alternative to shunt implantation [2, 3]. It has become preferred over a CSF diverting shunt, if correctly indicated, due to lower long-term complication and revision rates by avoiding foreign material implants [4–6]. Depending on the indication decision, approximately 30–35% of patients do not well respond to ETV and will require CSF shunting eventually [7]. This rate of success varies significantly among patients, influenced by a complex interplay of multiple factors [6, 8].

Correct assessment and evaluation of patient selection who might best benefit from ETV will improve patients’ safety and might significantly reduce overall medical costs. The current most utilized method to predict ETV outcome is a ETV success score (ETVSS), predicting the 6-month rate of shunt freedom [9]. It is basically based on the patients’ age, the underlying cause of hydrocephalus, and the previous state shunt treatment. It previously underwent internal [9] and external validation [10, 11] with good results in prediction value. However, a possible restriction in this scoring system might be that it does not include any radiological features, which are still relevant for daily surgical decision-making and have shown predictive value for ETV success as well [12–14].

In a recent systematic review and meta-analysis to assess the prognostic performance of bulging of the third ventricular floor (3^rd^VF) as a sign of the pressure gradient between the internal and external CSF spaces, bulging 3^rd^VF is associated with increased ETV success [15]. In addition, trans-ependymal periventricular edema was reported to correlate with a higher ETV success rate [16]. In a single-center study, the “Heidelberg ETV score” was introduced, which confirmed that the presence of a pressure gradient at the lamina terminalis and the 3^rd^VF correlates with avoiding ETV revision or shunt implantation after initial ETV [17]. Other radiological markers may also be relevant as predictors for ETV success such as the role of ventricular width in combination with signs of pressure gradient signs at the third ventricle was found to be relevant in prepontine occlusive hydrocephalus [18].

Our current study investigates specific MRI markers such as bulging of the third ventricular floor, lamina terminalis, and pineal recess, together with aqueduct stenosis, prepontine occlusion, fourth ventricle outflow obstruction, and frontal-occipital horn ratio (FOHR) in relation to ETV success for shunt freedom. Thereby, we are aiming to develop a predictive model on the basis of combined clinical and radiological criteria of a single-center retrospective case series.

## Methods

We collected data from all ETV procedures on patients performed in the pediatric Neurosurgery Department at Charité Universitätsmedizin, Berlin, Germany, between January 2010 and December 2019. All the patients were under 18 years of age and suffered from symptomatic hydrocephalus usually with MR imaging signs of non-communicating CSF circulation disturbances. Most patients received ETV as initial treatment, and some patients may have been previously treated with a CSF shunt, and ETV was performed to achieve shunt explantation. Patients’ follow-up time was at least 6 months. Successful ETV was defined as the patient remaining shunt-free for the entire duration of follow-up. Data collection was performed retrospectively according to the digital in-house surgical data documentation system. Local ethics committee approval was given (EA2/132/17).

### Clinical data acquisition

Each patient’s clinical course was recorded until the time of the last follow-up appointment at our institution. Possible revision surgeries such as revision ETV and shunt implantations were documented in order to calculate the revision-free survival. Patients were categorized according to ETV success score (ETVSS) with age, the cause of hydrocephalus, and previous history of ventriculoperitoneal (VP) shunt. The preoperative ETVSS was calculated for each patient [9]. Patients were divided into three strata according to their ETVSS [19]: high, ≥ 80; moderate, 50–70; and low, < 50. In addition, we specifically categorized age at intervention being ≤ 6 months or > 6 months as it was done previously in the IIHS study [20]. For diagnosis, we additionally distinguished between types of hydrocephalus associated with protein overload (post-hemorrhagic hydrocephalus, PHH), hydrocephalus caused by membrane-related obstruction at the level of pre-pontine cistern also described as prepontine occlusive hydrocephalus [18], and other types of underlying diagnosis.

### Radiological data acquisition

The availability of paired preoperative and postoperative images was the precondition for inclusion in the study. Preoperative and postoperative MRI studies were reviewed, and measurements were taken using the hospital-based picture archiving and communication system (PACS) images using the “measurement tool” (Phönix-PACS, Freiburg, Germany). The fronto-occipital horn ratio (FOHR) was calculated using the formula — “(Maximum frontal horn diameter + maximum occipital horn diameter) / 2 × the biparietal diameter” [21]. All measurements were performed on 3D MRI data, either T2- or T1-weighted, which were reformatted into axial slices angled parallel to the undersurface of the corpus callosum. In addition, radiological characteristics for non-communicating hydrocephalus were recorded. That included possible bulging of the third ventricular floor (3^rd^VF), the lamina terminalis (LT), and the pineal recess (PR), as well as obstruction of the Sylvian aqueduct (SA), the fourth ventricular outlets (4^th^VO), or at the prepontine cistern (PC). The evaluation was performed, and measurements were taken by DT, UWT, and AT. In cases of possible mismatch of evaluations, a consensus decision was made by reviewing the images of the case together, respectively.

The presence of a pressure gradient between internal and external CSF spaces was analyzed for each anatomical structure of 3^rd^VF, LT, and PR independently. Similarly, for outflow obstruction in each structure, the SA, 4^th^VO, and PC were evaluated separately. FOHR was categorized into two groups: low FOHR, ≤ 0.56; and high FOHR, > 0.56, according to the logistic regression model.

### Statistical analysis

Kaplan–Meier analysis was performed to evaluate ETV success, defined as remaining shunt-free throughout the patient’s follow-up period. The event was considered to be the placement of a VP shunt, and time to event was measured from the date of the initial ETV surgery to the date of the first shunt implantation. A categorization for FOHR was based on a logistic regression model applied to FOHR alone for the absence of implantation of a subsequent VP shunt during follow-up. The corresponding graph (see supplemental material) showed its peak at a FOHR equal to 0.56 distinguishing between patients with high or low FOHR. A log-rank test was used for the Kaplan–Meier analysis to compare shunt-free survival rates between two FOHR and age categories. Fisher’s exact test was used to assess the statistical significance of each dichotomized variable for possible relative differences of patients remaining shunt-free. Odds ratios and confidence intervals were calculated using the Baptista-Pike method. Statistical significance was considered with a *P* value < 0.05.

A multivariate predictive model was made using multiple logistic regression model starting with ten independent variables (dichotomized: age, 3^rd^VF, LT, PR, presence of pressure gradient, occlusion at SA, 4^th^VO or PC, category of HC diagnosis, and existence of a shunt before ETV, and continuous: FOHR) and applying the backward elimination technique [22] (elimination criteria *P* > 0.15, similar to previous publications [9]). Multicollinearity was assessed with variance inflation factors (VIF), to assess a single predictor variable as a linear combination of the remaining predictor variables. Multicollinearity is present when the VIF is higher than 5 to 10 [23]. Variables that had a linear connection with other variables were excluded to successfully run the logistic regression.

All analysis was performed with GraphPad Prism version 10.1.1 (GraphPad Software, Boston, MA, USA). Additional information is given in supplemental material.

## Results

A total of 136 patients with hydrocephalus were treated with ETV during the defined time period. After applying the defined inclusion criteria, the study included 95 patients in total (Table 1). The average time of follow-up was 38.4 ± 30.24 months. The mean patients’ age at the time of ETV was 88 ± 9.8 months. Seventy of 95 (74%) children remained shunt-free after ETV during the follow-up.

**Table 1:** Patients’ characteristics

Variable	Total sample
No. of patients operated (2010–2019)	136
Patients excluded due to	
Missing pre- or post-surgery MRI	28 (21%)
< 6-month FU time	9 (7%)
Aim of surgery was not shunt freedom	4 (3%)
Final number of patients included in the study	**95 (41%)**
No. of patients with VP shunt at follow-up	**25 (26%)**
No. of patients needed re-ETV	**13 (14%)**
Age at ETV	
< 1 month	1 (1%)
1 to < 6 month	8 (8%)
6 to < 12 month	11 (12%)
1 to < 10 years	47 (49%)
> 10 years	28 (29%)
Cause of hydrocephalus	
Aqueductal stenosis	35 (37%)
Brain tumor (non-tectal)	23 (24%)
Post-hemorrhagic	5 (5%)
Prepontine occlusive hydrocephalus	17 (18%)
Other	15 (16%)
Previous CSF shunt in place	5 (5%)

**Fig. 1:**
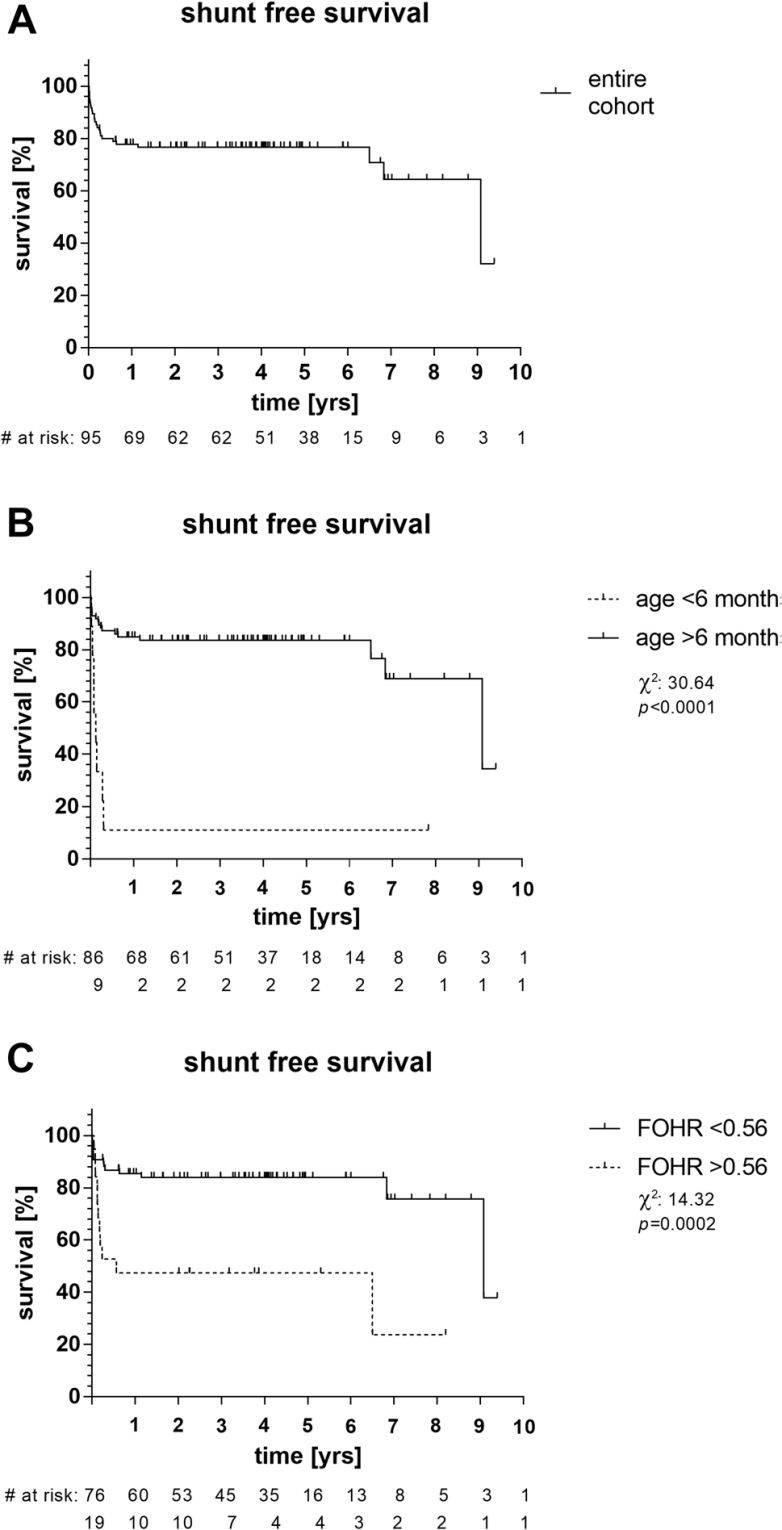
Kaplan–Meier survival curve for study cohort describing shunt independence over time. **A** For the entire cohort one-year survival revealed 77.8% and 5-year survival is 76.6%. **B** Comparing the patients according to age groups the survival differed in the older children > 6 months versus infants with < 6 months of age (1 year, 84.8% vs. 11.1%; 5 years, 83.5% vs. 11.1%; *χ*^2^ = 30.64,* P* < 0.001). **C** Looking at dichotomized FOHR groups (> 0.56 vs. < 0.56), a significant difference in shunt-free survival could be observed (1 year, 85.4% vs. 47.4%; 5 years, 83.9% vs. 47.4%; *χ*.^2^ = 14.32, *P* < 0.001)

The Kaplan–Meier survival curve shows shunt-free survival rates in the entire cohort (Fig. 1A) and within two age and FOHR groups (Fig. 1B, C). Comparison between two groups showed that the mean estimated shunt-free survival in the high FOHR (> 0.56) group was 3.18 years, compared to 7.47 years in the low FOHR (< 0.56) group. The log-rank test has shown a significant difference as well between the two age and FOHR groups (age: *χ*^2^ = 30.64, *P* < 0.001; FOHR: *χ*^2^ = 14.32, *P* < 0.001).

The different ETV success rates according to the different variables are given in Table 2. Among those, age > 6 months (*P* < 0.001), pre-ETV FOHR ≤ 0.56 (*P* = 0.001), and PHH (*P* = 0.016) were significant. Looking at the radiologic variables separately, no statistically significant factor could be detected. Odds ratios between patients included in one subgroup compared to the other subgroup were highest for age > 6 months (OR [95% CI], 32.47 [4.83–363.9]), followed by non-PHH (OR [95% CI], 13.14 [1.92–162.5]) and low FOHR (OR [95% CI], 6.09 [2.16–16.33]).

**Table 2:** Predictors for determining ETV success

Variable	Number of patients with this parameter (%)	Percentage of patients with no subsequent VP shunt	Odds ratio for no subsequent VP shunt (95% CI)	*P* value
Age at ETV				
> 6 month	86 (91%)	80%	32.47 (4.83–363.9)*	< .001
Radiologic findings before ETV				
Low FOHR	76 (80%)	82%	6.09 (2.16–16.33)*	0.001
Bulged 3^rd^VF	69 (73%)	75%	1.44 (0.51–3.92)	0.598
Bulged LT	77 (81%)	75%	1.67 (0.51–4.94)	0.377
Bulged PR	61 (64%)	70%	0.65 (0.22–1.85)	0.608
Aqueduct stenosis	56 (59%)	77%	1.47 (0.59–3.60)	0.480
4^th^VO obstruction	28 (29%)	64%	0.53 (0.20–1.34)	0.210
PC occlusion	17 (18%)	71%	0.83 (0.23–3.39)	0.766
Clinical findings				
No PHH	90 (95%)	77%	13.14 (1.92–162.5)*	0. 016
No previous CSF shunt	90 (95%)	74%	1.94 (0.32–9.92)	0. 604

Total number of patients = 95. *P < 0.05

*ETV*, endoscopic third ventriculostomy; *VP shunt*, ventriculoperitoneal shunt; *FOHR*, fronto-occipital horn ratio; *3*^*rd*^*VF*, third ventricular floor; *LT*, lamina terminalis; *PR*, pineal recess; *4*^*th*^*VO*, fourth ventricle outflow; *PC*, prepontine cistern; *PHH*, post-hemorrhagic hydrocephalus; *CSF*, cerebrospinal fluid

According to the multivariate logistic regression analysis, the final model to predict ETV success contained four variables: age (< 6 months; *P* = 0.004), smaller FOHR (*P* = 0.01), absence of a 4^th^VO obstruction (*P* = 0.06), and bulging of LT (*P* = 0.03; Table 3). The area under the curve (AUC) was 0.8082, indicating a good discrimination for a model [24]. An unpaired *t*-test showed a statistically significant difference between predicted values for patients with successful and unsuccessful ETVs (*P* < 0.0001), with the difference in means, 0.3315 ± 0.0492.

**Table 3:** Logistic regression model for predicting ETV success

Variable	Parameter estimate	Odds ratio for no subsequent VP shunt (95% CI)	*P* value
Age at ETV			
> 6 month	3.376	29.25 (4.08–605.8)	0.0037
Radiologic findings before ETV			
FOHR	− 10.47	2.83 × 10^−5^ (4.7 × 10^−9^–0.045)	0.0094
4^th^VO obstruction	− 1.187	0.31 (0.09–1.015)	0.0558
Bulged LT	1.524	4.59 (1.15–19.6)	0.0324

Variance inflation factors < 1.3. The mean predicted probability of shunt independence (*n* = 70) was significantly higher than in those who got one (*n* = 25) (difference between means [95% CI], 0.3315 [0.2338–0.4292], *P* < 0.0001). *C* statistic, 0.8082. The Hosmer–Lemeshow statistic was not significant (*P* = 0.7905), suggesting adequate model fit. Nagelkerke’s *R*^2^, 0.4049

*ETV*, endoscopic third ventriculostomy; *FOHR*, fronto-occipital horn ratio; *4*^*th*^*VO*, fourth ventricular outflow; *LT*, lamina terminalis

**Fig. 2:**
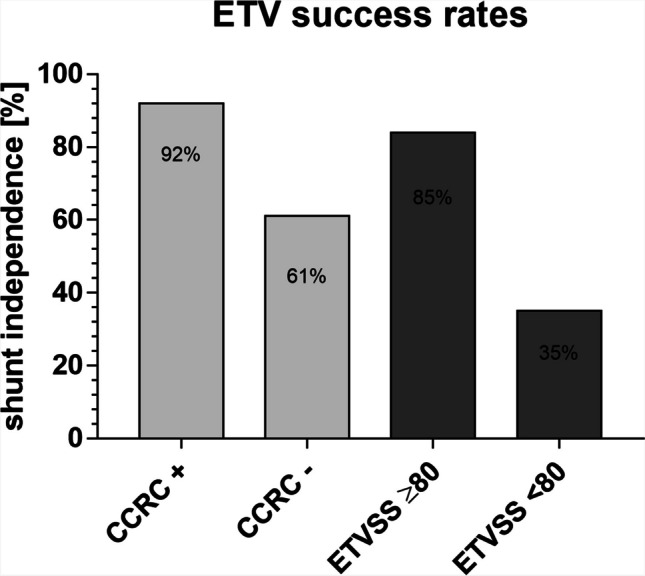
Rates of shunt independence after ETV are given for the combined clinical and radiologic criteria as analyzed in this study (CCRC + : age > 6 months and FOHR < 0.56 and bulged lamina terminalis and no obstruction at 4.^th^ ventricular floor) and the ETV success score (ETVSS) being ≥ 80 or < 80. The ETV success rate of patients from our cohort remaining shunt-free during follow-up was 92% in patients meeting all CCR criteria while it was 61% in patients in which at least one factor did not fit. In comparison, the success rate was 85% in the same cohort when ETVSS was ≥ 80 and 35% when ETVSS was < 80. (Abbreviations: CCRC + , patients who meet all four criteria; CCRC − , patients who do not meet one or more of the criteria; ETVSS ≥ 80, patients with a ETV success score above or equal to 80; ETVSS < 80, patients with a ETV success score lower than 80)

In the current cohort, the difference in actual success rate among patients who meet combined clinical and radiological criteria of age and radiologic sign was slightly higher (92%) compared to the success rate with high ETVSS of over 80 (84%; Fig. 2).

## Discussion

Although ETV is a relevant surgical option for non-communicating hydrocephalus, the primary challenge has been how to predict its success and how to identify patients who will most likely benefit from this procedure. The model developed in this study integrates key radiologic variables, which are relevant for eligibility and may influence the outcome of ETV, thereby helping surgeons in decision-making.

Similar to previous studies [9, 19, 25–28], our study has shown that age is one of the most important predictors of ETV success. Patients younger than 6 months seem to have the highest risk of ETV failure necessitating subsequent VP shunting. On the other hand, our study could not confirm that the radiological presence of aqueduct stenosis and absence of aqueductal flow void is an independent predictor of ETV success as suggested previously [9, 19, 28–30]. Similarly, in the independent evaluation, neither the obstruction of the prepontine cistern nor the 4th ventricular outflow showed to be of predictive value for success. In fact, 4th ventricular outflow obstruction was rather a negative predictive factor in our cohort according to the logistic regression model. Finally, it was not possible to combine all obstruction points into a single parameter with at least one location being blocked, as nearly all patients in our cohort showed at least one obstruction.

The FOHR is a measurement used in neuroimaging to assess ventricular width, especially in pediatric patients with hydrocephalus [31, 32]. Previous studies have demonstrated a significant FOHR reduction after ETV [18], which can be interpreted as a surgical success, as a decrease in ventricular size may improve brain development [33, 34]. However, FOHR has not been previously utilized for predicting the success of ETV so far. Our study shows that patients with a low FOHR (≤ 0.56) had a better chance of avoiding subsequent VP shunt implantation compared to the high FOHR (> 0.56) group. Additionally, in the logistic regression model using FOHR as a continuous variable, smaller ventricular width strongly predicted shunt independence after ETV. This may be due to the correlation between ventricular size and chronicity of hydrocephalus, suggesting that patients with more acute hydrocephalus may have better outcomes of ETV [35].

Unlike previous studies, we were unable to show that outward bulging of the third ventricular floor is an independent predictor for ETV success [15, 36, 37]. However, the bulging of lamina terminalis was a significant positive predictor in the logistic regression model. On the other hand, possible changes in the pressure gradient after surgery needs may serve as a post hoc indicator of a successful outcome, which needs further research [17].

The logistic regression finally defines the model used in our study, combining clinical and radiological factors to predict the likelihood of ETV success. The key predictors include age over 6 months, smaller FOHR, bulging of the lamina terminalis, and no obstruction at the 4th ventricular outlets. We found that patients who met all four criteria of our model had a higher likelihood of staying shunt-free compared to those with an ETVSS of ≥ 80 (92% vs. 84%, respectively). However, we also noted a significant difference in success rate between patients with high ETVSS and moderate/low ETVSS (84% vs. 35%, respectively) which corresponds to the results of previous studies [19]. This combined clinical and radiological criteria model may serve as a valuable tool in decision-making for pediatric neurosurgeons.

Possible limitations of this study include the limited number of patients collected in a retrospective analysis at a single institution. We are fully aware that robust conclusions for a valuable model would require validation in a multicenter cohort. Nevertheless, we were able to define a preliminary model on the basis of our cohort, which needs further investigation on a larger scale and may be refined with additional data. In addition, it needs to be emphasized that the indication for ETV and the selection criteria for patients included in any statistical model significantly influence the results. In our study, we performed ETV only in patients with radiological signs of obstruction between internal and external CSF spaces, consistent with non-communicating hydrocephalus. This also includes the fact that we successfully performed ETV in prepontine occlusive hydrocephalus, as described previously [18], which might be less common in other institutions. Similarly, we only included a limited number of post-hemorrhagic and no postinfectious patients in our study, which also differs from previous studies. This emphasizes the need for multicenter analyses with larger cohorts, where individual clinical decision-making may have less impact on results. Finally, our analysis does not precisely quantify the weight of each variable in the logistic regression model, which means that this model cannot be directly translated into a precise scoring system. Future studies, both retrospective and prospective, will be essential to validate these results and are likely to lead to refinements of the model.

In conclusion, we successfully tested a combined clinical and radiological model to predict the success of ETV, defined as shunt independence during follow-up. In this model, age, smaller ventricular width, and the presence of a pressure gradient at the lamina terminalis were identified as positive predictors for ETV success. The study should be considered a feasibility study for statistical modelling of ETV success using a combination of clinical and radiological criteria. Further investigation in a larger, multicenter patient cohort is necessary to validate and refine this model, aiming to develop a robust statistical tool for future use.

## Supplementary Information

Below is the link to the electronic supplementary material.

Supplementary file1 (DOCX 161 KB)

## Data Availability

No datasets were generated or analyzed during the current study.
