# Supplementary material for: Clinical and radiologic criteria to predict endoscopic third ventriculostomy success in non-communicating pediatric hydrocephalus
Source: Childs Nerv Syst. 2024 Dec 16;41(1):57. doi: 10.1007/s00381-024-06704-1 (PMC11649818; doi:10.1007/s00381-024-06704-1)
Supplement: Supplementary file 1 — Supplementary file1 (DOCX 161 KB) [file 381_2024_6704_MOESM1_ESM.docx]

SUPPLEMENTAL MATERIAL

*Statistical analysis*

The decision to divide patients in 2 FOHR groups at precisely at 0.56 was based on the proportion classified correctly on logistic regression model, when we ran it on only FOHR as variable and no subsequent VP-shunt as an outcome. Corresponding graph (see figure below) showed small pick at the point where FOHR = 0.56 that lead to our decision.


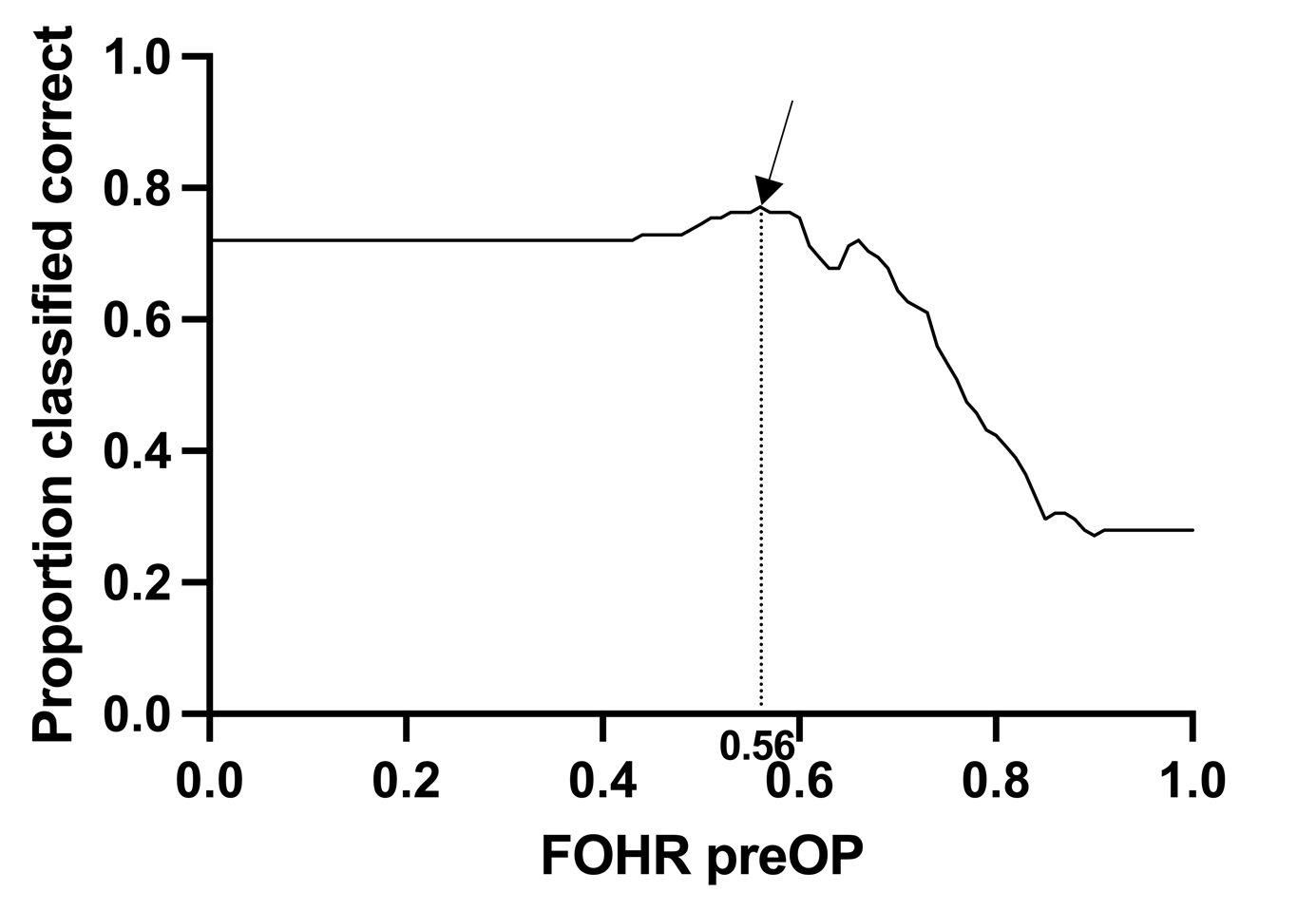


We tested the adequacy of final model as follows. Significancy of the model was assessed using Chi-square test for predicted and actual outcome. Goodness-of-fit was tested with Hosmer-Lemeshow statistic; a significant *p* value rejects the null hypothesis for the model fitting the data ^1^. Model discrimination was assessed by determining the area under the curve, which is equivalent to the C statistic^2^. This statistic can be interpreted as the probability that the model predicts a higher chance for ETV success in an actual successful case compared with failure; a value closer to 1.0 represents better model discrimination. We also compared the mean of predicted values between patients with and without subsequent VP-shunt.

Akaike's Information Criterion (AIC) is a statistical measure used to evaluate and compare the relative quality of statistical models for a given dataset. AIC assesses the trade-off between the complexity of the model (number of parameters) and how well the model fits the data. Lower AIC values correlates with better results^3^.

Variance inflation factor was <1.5 for each variable proving no multicollinearity. Hosmer-Lemeshow statistic was *P*=.7905 confirming good model fit. Mean of predicted values from the logistic regression model among all patients were 0.7312 ± 0.02656, which corresponds to actual success rate among our cohort – 0.75.

1. Hosmer D. A goodness-of-fit test of the multiple logistic regression model. *Commun Stat-Theory Methods - COMMUN Stat-THEOR METHOD*. 1980;9:1043-1069. doi:10.1080/03610928008827941

2. Hanley JA, McNeil BJ. The meaning and use of the area under a receiver operating characteristic (ROC) curve. *Radiology*. 1982;143(1):29-36. doi:10.1148/radiology.143.1.7063747

3. A new look at the statistical model identification | IEEE Journals & Magazine | IEEE Xplore. Accessed January 7, 2024. https://ieeexplore.ieee.org/document/1100705
